# Mechanism and Molecular Design Principles of Cationic Surfactants: From Charge‐Driven Membrane Interactions to Next‐Generation Quaternary Ammonium Compounds

**DOI:** 10.1002/cmdc.202501104

**Published:** 2026-03-13

**Authors:** Natalie Hanheiser, Yuhang Jiang, Chuanxiong Nie, Rainer Haag

**Affiliations:** ^1^ Institute for Chemistry and Biochemistry Freie Universität Berlin Berlin Germany

**Keywords:** bacterial resistance, cationic surfactant, membrane interactions, molecular design, quaternary ammonium compounds

## Abstract

Cationic surfactants, in particular quaternary ammonium compounds (QACs), represent one of the most relevant and broadly applied classes of antiseptics. Their antimicrobial activity arises from electrostatic interactions with microbial membranes, resulting in rapid disruption of the membrane structure. In this review, we summarize currently described mechanistic insights into the membrane active behavior of QACs, thereby focusing on the interplay between molecular architecture, supramolecular organization and antimicrobial efficacy. Key structure activity relationships (SARs) are discussed, including the role of the hydrophobic tail length, spacer design, charge density and distribution, and counterion effects. Addressing challenges such as antimicrobial resistance and biocompatibility requires a detailed understanding of SARs and the mechanism behind resistance development. Therefore, we further highlight emerging concepts such as cleavable linkers, hybrid systems integrating metal, peptide or photodynamic modalities, supramolecular aggregates, and the integration of biodegradable materials for the design of surfactants capable of overcoming bacterial resistance and tuning selectivity toward bacterial cells. This review provides an updated framework for developing next‐generation QACs that preserve antimicrobial potency while minimizing toxicity and the evolution of resistant microbial populations.

## Graphical abstract

Within this review we provide an overview of the social relevance of quaternary ammonium surfactants, different classes of these agents, their interaction mechanism with cellular membranes, structure activity relation, bacterial resistance toward these systems and new approaches to guarantee their long‐term efficiency.

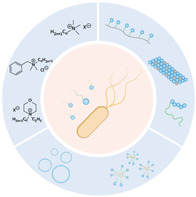

## 1 Introduction and Chemical Classification

The beginning of the 20th century is marked as the golden age of bacteriology. The groundbreaking work of Robert Koch, Paul Ehrlich, and Emil von Behring set the basis for modern hygiene and health standards and paved the way for modern medicine to develop [1]. Around the same time, Domagk was the first one to publish his pioneering work on the use of quaternary ammonium compounds as general and medical disinfectants [2]. Quaternary ammonium compounds (QACs or “quats”), formally described as NR_4_^+^ with R being an alkyl or aryl group, contain hydrophobic and hydrophilic units making them work as a surface active agent (surfactant) [3]. Since their initial discovery, the structure of QACs has been modified in different ways in order to fine tune their antibacterial activity, their biocompatibility, their cytotoxicity, and their chemical accessibility. Today, more than 326 QACs are known according to the European Inventory of Existing Commercial Substances [4]. Based on their structural motifs QACs can be separated into different categories: (i) Alkyl and hydroxyalkyl substituted QACs, (ii) nonhalogenated benzyl substituted QACs, iii) di‐ and trichlorobenzyl substituted QACs, and (iv) QACs with unusual substituents [5]. Among all QACs, cetyltrimethylammonium bromide (CTAB) [6], cetylpyridinium chloride (CPC), [7] and benzalkonium chloride (BAC) [8] are the most prominent representatives.

In 1985, QACs were patented by the Henkel AG & Co.KGaA for their use in haircare products [9]. Besides their use in the cosmetic industry, QACs are widely used as biocidal products for example in medicine, veterinary medicine, and the food industry [10]. During the COVID‐19 pandemic, the overall use of QACs increased drastically [11]. However, the overuse and misuse of these agents resulted in an increase in antimicrobial resistant bacteria strains [12]. Thereby, bacterial resistance to QACs can be intrinsic, through the formation of biofilms and bacterial endospores, or acquired through genetic mutations [13]. In comparison to antibiotics, the mechanism by which disinfectants destroy bacterial cells is unspecific as they act on multiple cell targets. Therefore, their destructive properties are not limited to bacterial cells resulting in a cytotoxic effect, e.g., toward mammalian cells. This cytotoxic effect can result in harmful skin irritations and even the development of chronic skin disease [14, 15, 16, 17, 18]. Specifically, the overuse of QACs in agriculture and livestock farming resulted in an increased exposure of these substances to the aquatic system where they cause an imbalance of the ecological system [19]. This review aims to give an overview of the different types of QACs, their mechanism of action, their structure activity relationship, bacterial resistance development to QACs, and new trends for the development of new QACs.

Quaternary ammonium compounds (QACs) can differ in several structural motifs, including (i) variation of the substituents (R groups), such as alkyl chains of different lengths or aromatic rings; (ii) the number and structure of quaternary ammonium cations; and (iii) the nature of the counter anion (Table 1).

**TABLE 1:** Overview of different groups of QACs.

Group	Subgroup	Structure	Example	Ref
Alkyl‐substituted QACs	Alkyl trimethylammonium compounds (ATMACs)	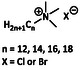	Cetyltrimethylammonium bromide	[20]
	Dialkyl dimethylammonium compounds (DADMACs)	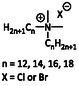	Dioctyldimethylammonium bromide	[21]
	Tetradecyltrimethylammoniumoxalate (CHPTAC)	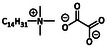	Handelsname Cadrimox‐14	[22, 23]
Non‐halogenated benzyl substituted QACs	Benzylalkyldimethyl ammonium compounds (BACs)	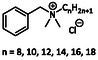	Benzyltrimethylammonium chloride	[24]
	Alkyldimethylethylbenzyl ammonium compounds (EBACs)	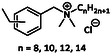	C12‐C14‐alkyl(ethylbenzyl)dimethylammonium chlorides	[25, 26]
Diand tri chlorobenzyl substituted QACs	—	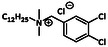	Lauryldimethyl‐3,4‐dichlorobenzylammonium chloride	[27]
QACs with unusual substituents	Alkyl pyridinium compounds	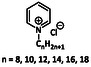	Cetylpyridinium chloride	[7]
	Imidazolinium compounds	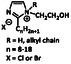	1‐Alkyl‐3‐hydroxyethylimidazolium chlorides	[7, 28]
	*N‐*EthylMorpholinium	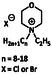	Decyl‐Ethyl‐Morpholinium	[7, 29]
	Esterquats	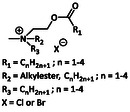	*N*,*N*,‐dimethyl‐3‐aminopropane‐1,2‐diol	[30]
	Polyquaternium compounds (polyquats)	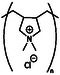	Polyquat‐1 Polyquat‐6	[31]
	Gemini surfactants	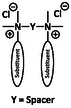	α, ω‐polymethylene‐bis(*N*‐octylpyridiniumbromide)	[32]

In terms of regulatory classification, QACs are commonly categorized according to the system defined by the United States Environmental Protection Agency [19, 33, 34, 35]. The largest and most widely used group of QACs includes those with alkyl substituents. Compounds containing three methyl groups and a single long alkyl chain are known as alkyltrimethylammonium compounds, such as cetyltrimethylammonium bromide (CTAB), a detergent widely used in microbiology for DNA extraction [20]. QACs with two long alkyl chains and two methyl groups are referred to as dialkyldimethylammonium compounds (DADMACs), also described as double‐chain cationic surfactants [21]. A subgroup of long‐chain QACs contain oxalate counterions, such as tetradecyltrimethylammonium oxalate (CHPTAC), which is frequently used in RNA extraction protocols [22, 23].

Another major QAC class comprises compounds containing a benzyl ring, a long alkyl chain, and two methyl groups. These benzylalkyldimethylammonium compounds (BACs), such as benzyltrimethylammonium chloride, are widely used as disinfectants and cleaning agents because of their bactericidal, fungicidal, and virucidal properties [24]. A structurally related subgroup incorporates an additional ethyl substituent on the benzyl ring, forming alkyldimethylethylbenzylammonium compounds (EBACs). EBACs such as C12–C14‐alkyl(ethyl benzyl)dimethylammonium chlorides are commonly used in sanitizing solutions and liquid soaps [25, 26].

QACs with chlorinated benzyl rings are accounted to the group of di‐and tri chlorobenzyl substituted QACs with lauryldimethyl‐3,4‐dichlorobenzylammonium chloride being the so far only representative. It is also used as a non‐oxidizing biocide for water disinfection and for the control of algae and biofilm formation in industrial water systems [27].

The final major category includes QACs with unusual heterocyclic or functional substituents. This group includes compounds in which the quaternary ammonium nitrogen is integrated into heterocyclic scaffolds such as pyridinium (alkylpyridinium compounds), imidazolinium, *N*‐ethylmorpholinium, or isoquinolinium systems [7, 28, 29]. It also includes ester‐containing QACs (esterquats) and polyquaternary structures (polyquats) which contain multiple quaternary ammonium centers [30, 31].

Cationic gemini surfactants represent an emerging and promising class of quaternary ammonium compounds. Similar to dialkyldimethylammonium compounds, gemini surfactants contain two long alkyl chains. However, unlike DADMACs, each alkyl chain is attached to its own quaternary ammonium headgroup, and the two cationic centers are connected via a spacer group *Y* (e.g., an alkyl chain, ester, carbamate, or other linking unit) (Table 1) [28]. This structural arrangement induces an enhanced surface activity, tunable charge density, and unique self‐assembly behavior compared with conventional monomeric QACs [32].

## 2 Mechanism of Action

Antibiotics exert their effects by binding to specific biological targets such as RNA or DNA, transpeptidases, or metabolic enzymes. In contrast, cationic surfactants show a different interaction profile. Their activity emerges from nonspecific, electrostatic interactions with the cell envelopes and intracellular structures rather than from specific ligand‐receptor interactions. This multitarget interaction profile makes the development of classical resistance mechanisms more challenging and underlines the therapeutic relevance of cationic molecules (including cationic surfactants, quaternary ammonium compounds, and antimicrobial peptides). Despite their chemical diversity, the mechanism by which these agents interact with cellular membranes can be mainly distributed in two steps: (i) adhesion to the cellular membrane via electrostatic interactions and (ii) insertion into the cellular membrane. The efficiency of these steps depends on structural characteristics such as overall charge, distribution of hydrophobic moieties, steric effects, multivalency, and the ability to form supramolecular aggregates.

### 2.1 Influence of the Bacterial Envelope Architecture and Charge

The first determining factor of antibacterial selectivity is the composition and architecture of the cell envelope. The structure of the cell surfaces differs between Gram‐positive and Gram‐negative bacteria. Gram‐positive bacteria possess a thick, highly crosslinked peptidoglycan matrix (20–80 nm), enriched with teichoic acids (TAs) and lipoteichoic acids (LTAs). Both TAs and LTAs contain anionic phosphate and glycerol groups that contribute to the surface charge. Gram‐negative bacteria exhibit a more complex double‐membrane architecture. The outer membrane (OM) features an asymmetric bilayer with lipopolysaccharides (LPS) in the outer leaflet and phospholipids in the inner leaflet. LPS molecules contain lipid A, core oligosaccharides, and *O*‐antigen polysaccharides, which collectively contribute to the negative charge of the cell surface. Below this lies the periplasm and a thinner peptidoglycan layer, followed by the inner membrane [36].

Zeta potential measurements consistently report values between approximately –5 and –35 mV for diverse bacterial species, confirming the uniformly anionic nature of the microbial envelope [37]. This negative charge most likely arises from the negatively charged lipids, such as phosphatidylglycerol (PG) and cardiolipin (TMCL). The negative charge enables cationic molecules to associate with the cell surface in a thermodynamically favorable manner [38].

In this regard, an interesting approach was discussed by Multhaup et al. who describe how the charge ‐ driven interactions of proteins and DNA or RNA is driven by counterion release. Protein patches with positive charge can interact with negatively charged polyelectrolytes. Thereby the proteins become multivalent counterions of the polyelectrolyte. The replacement of the monovalent counterion through a multivalent counterion (protein) results in a gain in entropy which is the main driving force for the charge ‐ driven binding between the polyelectrolyte and the protein [39]. Similar to that, we propose that the interaction between positively charged surfactants and the negatively charged bacterial membrane is also driven by a gain in entropy resulting from the replacement of the monovalent counterions of the cationic surfactant through a multivalent counterion (bacterial membrane).

### 2.2 Mechanism of Membrane Interactions

**FIGURE 1:**
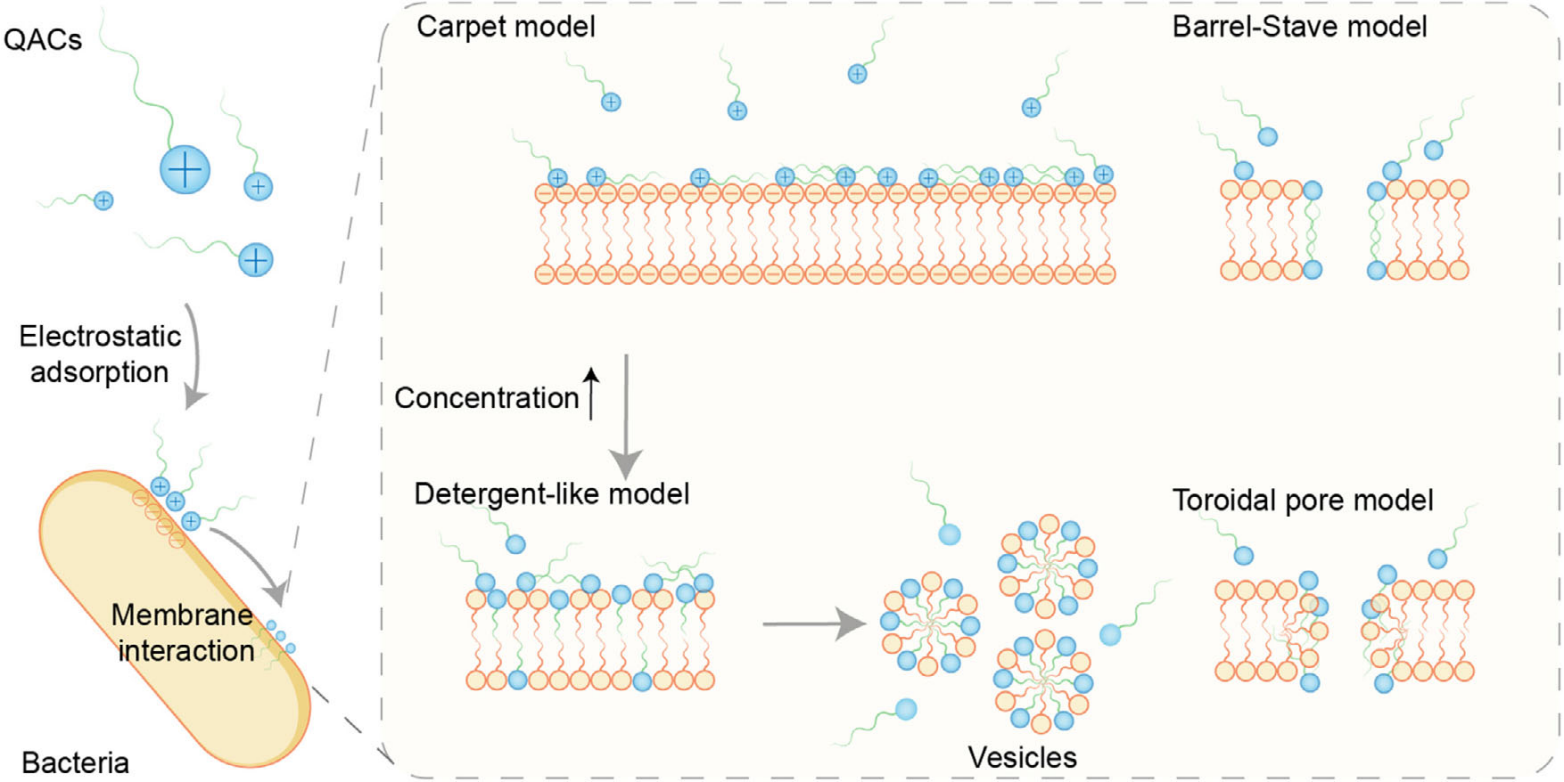
Schematic diagram of the interaction between QACs and bacterial membranes.

Once bound to the surface, cationic molecules undergo hydrophobic insertion into lipid bilayers. Several mechanistic models have been proposed to describe the diversity of membranolytic processes initiated by such molecules: (i) Barrel‐Stave, (ii) Toroidal pore, (iii) Carpet, and (iv) detergent‐like model (Figure 1).

I. The Barrel ‐ stave model primarily applies to cationic antimicrobial peptides with an amphipathic structure, which adopts an alphahelical conformation upon membrane contact. Helices insert perpendicularly into the hydrophobic membrane to form barrel‐like pores that are exclusively lined by peptide monomers [40].
II. According to the toroidal pore model the peptide adheres to the lipid head groups in the outer membrane and sticks to it. Above a certain peptide/lipid concentration, the peptide causes bending of the lipid‐bilayer such that lipids and peptides align in the pore interior. The formation of toroidal pores appears to be a concentration dependent process [41].
III. The carpet model describes the adsorption of cationic molecules parallel to the membrane surface, covering it like a “Carpet.” At a threshold concentration, the membrane breaks into smaller fragments.
IV. The detergent like model describes the continues lipid solubilization into mixed micelles caused by detergent‐like molecules. It is based on the fact that amphiphilic (macro)molecules and lipid bilayers have a similar chemical structure. Compounds that follow this mechanism of action are often used for membrane protein extraction. This process typically proceeds via three steps: (i) the vesicle range, in which the amphiphilic molecule position into the bilayer; (ii) the coexisting range, where both mixed micelles and vesicles occur; and (iii) the micellar range, where all membrane vesicles are fully dissolved and only micelles of varying lipidamphiphile composition are left [42].

Even though most of them are prone to describe interactions between cationic peptides and cellular membranes especially the carpet model, the toroidal pore and the detergent‐like model are most likely to apply for QACs.

### 2.3 Theoretical Evidence and MD Simulations

High‐resolution simulations have provided mechanistic insights into how cationic surfactants traverse complex bacterial cell walls. Using molecular dynamic (MD) simulations Sharma et al. demonstrated that monomeric surfactants could diffuse through the peptidoglycan layer, whereas micellar aggregates cannot. Their translocation efficiency depends on their molecular design (balance between hydrophobicity and charge distribution) and is influenced by the interplay between the surfactants and sugars/amino acids in the peptidoglycan layer. Within lipid bilayers, simulations reveal that cationic amphiphiles induce a disruption of the lipid packing resulting in a compromised membrane integrity. Compromising the membrane integrity can involve different mechanisms. Briefly, they found that membrane solubilization is induced by pore formation. Pore formation is initiated by the reorientation of the lipid and surfactant molecule to form a stable water pore. Ultimately resulting in membrane solubilization [43].

Experimental studies corroborate these computational insights. Ferreira et al. reported on the interaction profile of QAC benzyldimethyldodecylammonium chloride (BDMDAC) and the cell membrane of Gram‐negative *P. fluorescens*. Within their studies, they found that BDMDAC interacts with cellular membranes via ionic and hydrophobic interactions. Using zeta potential measurements, they found that treatment with BDMDAC changes the surface charge of *P. fluorescens* to less negative values, thereby indicating adhesion of the QAC to the cellular membrane. Live/dead staining using propidium iodide (PI) showed PI uptake in treated cells suggesting that BDMDAC treatment seriously compromises the integrity of the cytoplasmic membrane. Further, potassium cation leakage occurred after QAC treatment and increased with increasing QAC concentrations consistent with substantial cytoplasmic membrane damage. Interestingly, they found that cell‐BDMDAC interactions resulted in a change of the cell surface hydrophobicity with untreated cells presenting hydrophilic properties and an application of BDMDAC resulting in a decrease of hydrophilic characteristics and the acquisition of polar properties. Based on these results, they hypothesized that there is a specific binding receptor for QACs. Further, they suggested that QACs passage the outer membrane via different routes depending on their chemical nature. The hydrophilic route describes the entrance of hydrophilic QACs to the cytoplasm utilizing porin channels in the outer membrane, while the hydrophobic route describes the entrance of hydrophobic QACs via the disturbance of lipid fractions in the outer membrane. Within their studies, they evaluated the influence of BDMDAC on enzymes embedded in the cytoplasmic membrane. Using 1‐D electrophoresis, they could not find an alteration in outer membrane protein (OMP) expression which shows that BDMDAC exerts an antimicrobial effect without inducing the expression of resistant proteins at the outer membrane. Scanning electron microscopy (SEM) images were used to detect structural modifications in the physical appearance of the bacterial cells. In this, they found that BDMDAC treatment resulted in a deformation of the cellular membrane and a shrinking of the bacterial cells proving that cell‐BDMDAC interactions resulted in the release of intracellular material [44]. In line with the results from Ferreira et al., our group reported on the interaction of cationic surfactants and cellular membranes of Gram‐negative bacteria. In line with what is already described in literature, we found that the addition of cationic surfactant to the negatively charged bacteria resulted in a decrease in the negative charge of the bacteria indicating that this charge neutralization results from electrostatic interactions between the surfactant and the cellular membrane. Further, we used Cryo‐TEM images to gain a closer depiction of the cellular membrane before and after the treatment with the cationic surfactant. Within their studies, they found that the treatment with the cationic surfactant resulted in significant membrane disruption and finally to cell lysis [45].

### 2.4 Intracellular Effects

Although membrane disruption represents the primary mode of action, cationic amphiphiles can exert secondary intracellular effects once they access the cytoplasm. Interestingly, Inácio et al. found that cytotoxicity to mammalian cells occurs before membrane disintegration and cell lysis were observed. Within their studies, they found that QACs can induce inhibition of the NADH‐ubiquinone oxidoreductase and slowing down of the mitochondrial phosphorylation system, thus resulting in the fragmentation of mitochondria, a decrease in the mitochondrial transmembrane potential, a decrease in the mitochondrial volume, and an increased production of reactive oxygen species (ROS) [46].

### 2.5 Interactions with Fungal Cell Membranes

Fungal membranes differ substantially from bacterial membranes because their cell wall mainly contains chitin [47]. In their studies, Vieira et al. demonstrated that QACs interact with fungi primarily through charge shift from negative to positive rather than membrane solubilization. Interestingly, antifungal activity occurred below the CMC, implying that the monomeric species is responsible for initial interactions. Mammalian cells were more sensitive than fungi, likely due to differences in sterol composition and membrane fluidity. These findings highlight that QAC mechanisms are organism‐specific and depend critically on membrane composition [48].

## 3 Structure Activity Relationship

**FIGURE 2:**
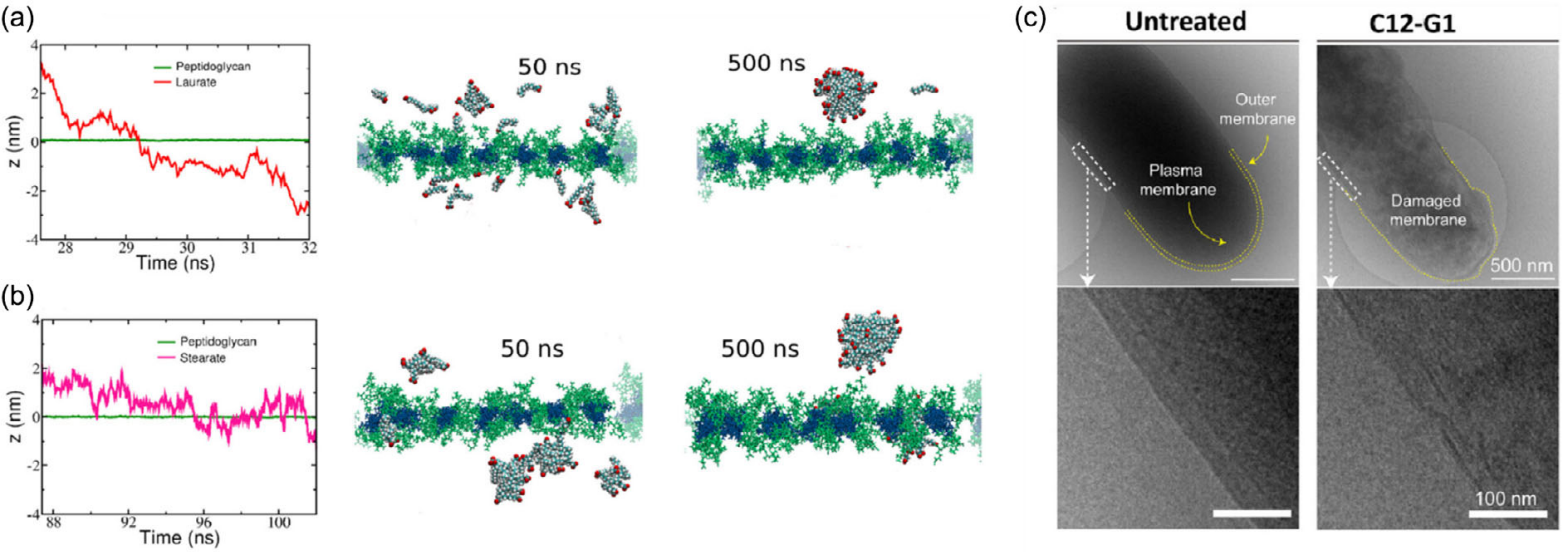
(a,b) Experimental evidence for interactions between QACs and bacteria cell membrane. Adapted from [43], Copyright 2022, Langmuir. (c) Cryo‐TEM images of bacteria cells before and after treatment with cationic surfactants. Adapted from [49], Copyright 2025, Angewandte Chemie.

**FIGURE 3:**
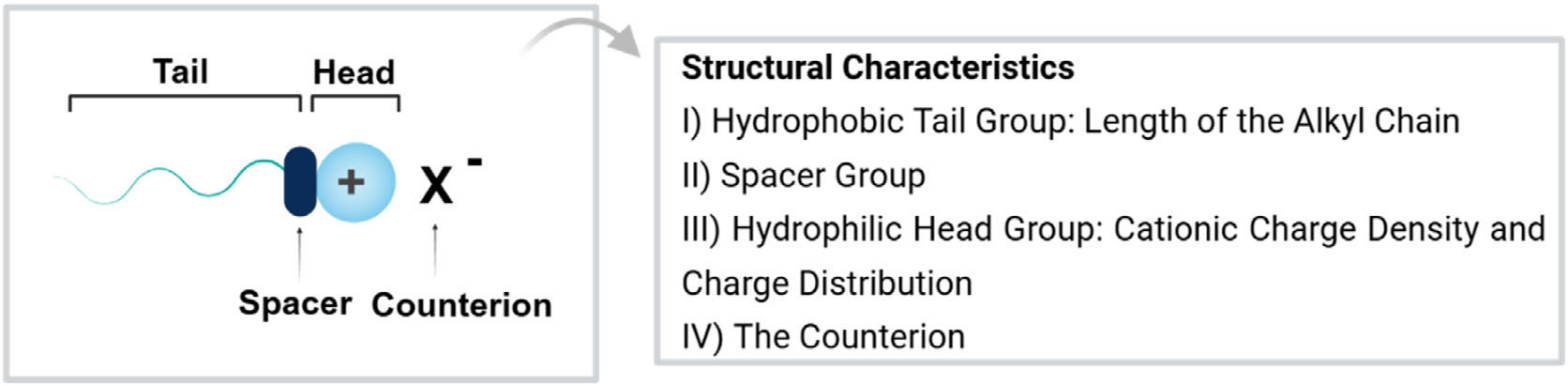
Structure activity relationship. Surfactant structure showing different parameters that alter the antimicrobial effect of these compounds.

Quaternary ammonium surfactants (QAS) represent one of the most structurally tunable classes of membrane‐active antimicrobials. Their molecular architecture can be divided into four major components: (i) the hydrophobic alkyl chain, (ii) the spacer or linker between head and tail, (iii) the cationic charge density and distribution, and (iv) the counterion (Figure 2). Additionally, supramolecular effects may influence their structure activity relationship. Each of these structural attributes governs how QAS interact with bacterial membranes, thereby shaping both their antibacterial potency and their cytotoxicity toward mammalian cells. The following sections summarize key structure–activity relationships (SARs) reported in the literature (Figure 3).

### 3.1 The Hydrophobic Alkyl Chain (Tail Group)

The alkyl chain length is one of the most influential parameters that determines the antimicrobial potency. Numerous studies consistently show that increasing the hydrophobicity to a certain point enhances the ability of QAS to insert into lipid bilayers, thereby boosting bactericidal activity.

Guo et al. systematically varied the alkyl chain length of PAMAM‐based quaternary ammonium dendrons. Their results demonstrated a direct correlation between hydrophobicity and antimicrobial potency. For example, for G1 PAMAM dendrons, no antibacterial activity was found for dendrons with a C6 alkyl chain, while the dendron with a C12 alkyl chain was moderately active, and the dendron with a C18 alkyl chain showed the highest antibacterial activity. Their findings support the hypothesis that longer hydrophobic chains facilitate stronger disruption of bacterial membranes if a proper balance between the hydrophobic and hydrophilic unit is kept [50].

Bustelo et al. observed a similar trend. The antibacterial activity strongly depends on the length of the alkyl chain. Within their studies, they compared single chain histidine‐based surfactants with a C10, C12, C14, or C16 alkyl chain with one another and found that the surfactant with a C14 alkyl chain was the one with the best antibacterial activity [51].

Zhang et al. investigated the role of the alkyl chain length in gemini quaternary ammonium surfactants on their antibacterial activity and cytotoxicity in vitro. Within their studies, they found a stronger antibacterial activity against Gram‐positive *S. aureus* than Gram‐negative *E. coli*, a clear enhancement of the antibacterial effect with an increasing chain length and C16 gemini QAC being the most active candidate. They attribute the Gram‐positive selectivity to structural differences in the outer membrane. Gram‐positive bacteria have a thicker but more porous peptidoglycan layer, while Gram‐negative bacteria have dense LPS layers making them less accessible for hydrophobic agents. Unfortunately, the hydrophobic tail that inserts into bacterial membranes also disrupts mammalian membranes at similar concentrations. Interestingly, Zhang et al*.* noted that their gemini QACs were less cytotoxic than classical monomeric surfactants such as CTAB or DTAB, suggesting that molecular symmetry and charge distribution can modulate selectivity [52]. Using molecular dynamic (MD) simulations, Makki et al. found that a flexible alkyl chain promotes ion exchange [53]. From this, we could conclude that a flexible alkyl chain supports binding of the surfactant to the bacterial membrane.

### 3.2 The Spacer Group

The spacer or linker between charged headgroups (in gemini surfactants) plays a crucial role in determining molecular flexibility, charge distribution, and accessibility of hydrophobic tails.

Zhang et al. further investigated the effect of the spacer group on the antibacterial effect of quaternary ammonium gemini surfactants. Interestingly, they found that the cationic charge density decreases with the rise of the spacer group. For the gemini surfactant with a C12 alkyl chain, it can be observed that the antibacterial effect increased with a moderate spacer length between C4‐C8, while very short spacers C < 4 and very long spacers C > 8 made the system less active. Surprisingly, the cytotoxicity decreased with a moderate spacer length [52]. The mechanistic details remain unknown so far.

With the aim to develop cationic surfactants that have a high selectivity toward bacterial cells over mammalian cells, Hoque et al. developed quaternary ammonium surfactants with a different chain length spacer group and cleavable amide linkage between the head and tail group. They found that the antibacterial potency depends on hydrophilic‐hydrophobic balance of the cationic amphiphile. With an increasing spacer length from 2 to 6 carbon atoms, the antibacterial effect increased, while with an increase of the spacer length from 6 to 12 carbon atoms the antibacterial effect decreased again. Interestingly, the hemolytic activity of these cationic surfactants was 9–10‐fold higher than the corresponding MIC values. From this the authors concluded that the hemolytic activity is controlled by hydrophobic interactions, whereas for the antibacterial activity, charge‐driven interactions seemed to be more important [54].

Similarly, Brycki et al. investigated the relationship between antimicrobial activity and alkyl chain length or the nature of the spacer. They also found that quaternary ammonium surfactants are more effective against Gram‐positive bacteria than against Gram‐negative bacteria. Within their surfactant library, they identified the surfactant with a C12 alkyl chain as the most effective compound. In this, the compound with a C4 alkyl chain showed the highest MIC values. They hypothesized that the butyl chain might be too short to insert into the outer shell of the microorganism. In comparison to the surfactant with a C12 alkyl chain, those with a C18 alkyl chain showed a 4 times higher MIC value against bacteria and fungi. This decrease in the antibacterial activity at a certain alkyl chain length is described as the “cut‐off” edge by Devinsky, a pioneering researcher on the field of quaternary ammonium surfactant synthesis. Importantly, lowest MIC values were observed for compounds containing a C10‐C16 alkyl chain. Further, Brycki et al. investigated the role of the spacer group on the antibacterial activity by replacing the ether linker through an amine, an alkyl chain, or an aromatic ring. Their results revealed similar antibacterial activities for the gemini surfactants with an ether, amine, and alkyl chain linker. The introduction of an aromatic ring as a spacer does not result in a better antimicrobial activity. From this, one can conclude that the introduction of an electron rich group in the spacer does not result in better aggregation of the biocide to the bacterial membrane as no increase in the antimicrobial activity was observed. Importantly, the gemini surfactants showed a better activity than their monomeric counterparts [55]. Within their studies, Frolov et al. found that pyridine‐based bis‐QACs showed improved antibacterial properties in comparison to mono‐QAC systems. Further, their studies underline that the balance between hydrophobic and hydrophilic units is crucial to achieve an improved antibacterial effect [56].

### 3.3 The Cationic Charge Density and Distribution (Head Group)

Electrostatic interactions between positively charged QACs and negatively charged bacterial surfaces are the primary drivers of initial adhesion and membrane perturbation. Cella et al. hypothesized that introducing electron‐withdrawing or electron‐donating substituents near the quaternary nitrogen (e.g., phenyl or cyclohexyl groups) would increase charge density and thus antibacterial activity. Therefore, they attached either a phenyl‐ or a cyclohexyl‐ring to a quaternary ammonium cation. Further, they introduced either a C1‐C3 alkyl chain between the nitrogen atom and the aliphatic ring. As both rings have either an electron‐withdrawing or electron‐donating effect, they should alter the positive charge density on the nitrogen atom. According to this hypothesis the QACs having the electron‐donating substituent directly attached to the nitrogen atom should show the strongest antibacterial effect. Contrary to their hypothesis, charge density according to their definition had only a minor influence. Instead steric effects dominated and restricted membrane insertion of cationic surfactants to cellular membranes [57].

In contrast, Colomer et al. correlated the charge density to the number of positively charged functional groups in the molecule. They evaluated three families of lysine‐based QACs with: (i) single cationic headgroups, (ii) dual amino‐acid‐derived headgroups, and (iii) gemini architectures with multiple charges.

They found that the antibacterial activity correlated with both number and position of positive charges and that arginine‐based QACs outperformed lysine‐based counterparts due to guanidinium groups which form stronger electrostatic interactions. Surprisingly, in their case, simple headtail structures were more effective than more complex gemini surfactants [58].

An interesting approach for the alteration of the antibacterial and cytotoxic effect was made by Pérez et al., who investigated different binary cationic mixtures containing a cationic histidine derived surfactant and an anionic surfactant. By modulating the cationic/anionic surfactant ratio in mixtures they were able to tune the cationic charge density and thereby the biological activity of the system. While the pure cationic histidine derivate exhibited a broad spectrum of antibacterial activity, the anionic surfactants showed no antibacterial activity. It showed that the antibacterial activity depends on the proportion of cationic surfactant and the tested microorganism. Overall, no activity of histidine based cationic surfactant alone and in mixture against Gram‐negative bacteria was found. Once the amount of cationic surfactant in mixture was ≤50%, the positive charge of cationic surfactants is neutralized, and the MIC values increased. The cationic surfactant alone showed to be cytotoxic. Notably, once the proportion of cationic surfactant decreased, the cytotoxicity decreased. Interestingly, the incorporation of cholesterol to the surfactant mixtures resulted in a reduced cytotoxicity of about 10%–30% [59].

This illustrates that charge density can be strategically tuned to optimize selectivity [60].

### 3.4 The Counterion

Although often overlooked, the counterion can affect solubility, membrane interactions, and reactivity. Most studies show that counterions exert a smaller influence than hydrophobic chain length. However, counterions can modulate biological activity by influencing the oxidation potential, steric coordination, solubility, and hydrophobicity of the cationic surfactants.

Kula et al. synthesized C12, C14, and C16 QACs paired with methyl carbonate, acetate, or bromide anions, respectively. They observed that the activity of these surfactants followed classical trends. Surfactants were more effective against Gram‐positive bacteria than against Gram‐negative bacteria and compounds with 14 and 16 carbon atoms in the alkyl chain were more effective than the ones with 12 carbon atoms. For the antibacterial activity of the QAC they found that the type of counterion was not as significant for the activity of the compound as the length of the hydrophobic aliphatic chain. The activity of quaternary ammonium salts can be enhanced by reactive counterions that can oxidize proteins or lipids. Therefore, they found that QACs with an acetate or bromide in their structure were slightly more effective for the eradication of biofilms. Unfortunately, bromide most likely enhances mutagenic properties [61]. Similar findings were made by Yudovin–Farber et al. who replaced iodine counterion through nitrate and acetate anions within quaternary ammonium polyethyleneimine nanoparticles. They found similar activities for all three compounds with the acetate and the nitrate counterions resulting in a slight increase in the antibacterial activity of the system. Further, they stated that the counterion only affects the antibacterial properties of the QAC where it alters the solubility of the biocide. An interesting study was performed by Yan et al. who specifically focused on QACs with acetate based counterions with varying carbon chain length in the counterion. They synthesized a library of dodecyl/tetradecyltrimethylammonium salts with an acetate based counter ion having either a one, two, three, five, seven, or nine carbon atom length alkyl chain. They compared the QACs with different chain length acetate counterions for their bacteriostatic efficacy and found that the bacteriostatic efficacy gradually decreased with increasing the carbon chain length of counterions, especially if it exceeds six carbon atoms [62].

### 3.5 Supramolecular Effects

QACs and cationic surfactants readily assemble into micelles, vesicles and higher‐order aggregates. The supramolecular state critically influences antibacterial properties. Zhou et al. reported that cationic micelles interact with *E. coli* via a two‐step process, which is quite similar to that of the single molecules: (i) the integrity of the outer membrane of Gram‐negative *E. coli* is disrupted by electrostatic interactions, followed by (ii) the inner membrane is disrupted by the hydrophobic interactions of the hydrophobic carbon chain of the surfactant with the hydrophobic domains of the inner membrane, resulting in the leakage of cytoplasmic material [63].

### 3.6 Multicationic Systems

In this context, multicationic systems refer to small molecules or polymers that carry multiple positively charged quaternary ammonium groups. For low‐molecular‐weight compounds, increasing the number of cationic charges has often been linked to improved antibacterial activity compared with monocationic analogs. A well‐known example is cetylpyridinium chloride (CPC)‐derived compounds, commonly referred to as bis‐QACs. Linking two CPC units via a bisphenol A spacer has been shown to enhance antibacterial efficacy relative to the corresponding monocationic QAC. Moreover, the use of isocyanuric acid as a trivalent scaffold enables the attachment of three CPC units, resulting in trimeric QACs with further increased antibacterial activity compared to both mono‐ and bis‐cationic units [64, 65]. Similar observations have been reported for polymeric systems bearing multiple (>10) quaternary ammonium groups. Increasing the molecular weight of the polymer, and thus the overall charge density, generally leads to stronger inhibition of planktonic bacterial growth [60]. Nevertheless, the assumption that a higher number of positively charged functional groups invariably results in greater antibacterial efficacy should be treated with caution. Other structural factors, particularly the balance between hydrophilic and hydrophobic segments as well as the length of the hydrophobic alkyl chains, play an equally important role in determining antimicrobial activity [45].

## 4 Applications and Risks

Due to their amphoteric nature, surfactants are able to solubilize both hydrophilic and hydrophobic compounds. Therefore, they are widely used in personal care products such as soaps, shampoos, and detergents. Specifically, in the medicinal and pharmaceutical industry, cationic surfactants play an important role as transfection agents for gene therapy techniques, as disinfectants because of their antimicrobial activity, and in protein extraction. Similar to the interaction profile of cationic surfactants with cellular membranes, cationic surfactants interact with DNA. The binding of cationic surfactants to DNA follows a two‐step binding isotherm, in which one step is dominated by electrostatic interactions and the other by hydrophobic interactions. Upon binding of the cationic surfactant to the negatively charged DNA, the charge of the DNA is neutralized, resulting in a net positive charge of the DNA surfactant complex. One interesting compound in the context of gene therapy is the quaternary ammonium surfactant cetrimide. Cetrimide lyses the outer cell membrane while leaving the nuclear membrane intact [66]. Further, surfactants play an important role in the extraction of amino acids and proteins. Interestingly, anionic surfactants are used for the extraction of important small monomeric proteins such as cytochrome c, trypsin, and bovine serum albumin. Depending on the charge of the surfactant, extraction yields positively, negatively charged, and neutral amino acid derivatives. This observation underlines the significance of electrostatic interactions between surfactant micellar aggregates and the extracted amino acids. CTAB is one cationic surfactant that is predominantly used for protein extraction [67]. Among all cationic surfactants, benzalkonium, dialkyldimethylammonium, and alkyltrimethylammonium compounds are most commonly used in disinfectants in the healthcare and food industry [68, 69]. Polyquats are often used in personal care products. Due to their positive charge, they neutralize anionic surfactants in shampoos and negatively charged proteins in the hair [70]. With regard to their application as disinfectants the cytotoxicity of these agents can result in harmful skin irritations [14, 15, 16, 17]. Thus, the cytotoxic effect of these agents remains an ongoing challenge which compromises their application spectrum. In response to the COVID‐19 pandemic the use of QACs in disinfectants has accelerated drastically resulting in an increased exposure to humans drawing attention to adverse ecological and health outcomes connected to QAC usage. Besides their toxic effect to the dermis and the respiratory system, QACs can result into development and productivity issues and the disruption of metabolic functions such as lipid homeostasis. Adverse ecological effects result from the contamination of the environment and lead to acute toxicity to the aquatic system. QACs have been found to promote the further development of antimicrobial resistance. Therefore, restrictions regarding the use of QACs, primarily in personal care products, have been implemented in the US [19].

## 5 Bacterial Resistance to Quaternary Ammonium Compounds (QACs)

The public health risks posed by multidrug‐resistant bacteria continue to escalate globally, with their dangers no longer being confined to hospital settings but increasingly spreading in outpatient and industrial environments [71, 72]. Over recent decades, quaternary ammonium compounds (QACs) have been extensively employed as disinfectants and preservatives in hospitals, cosmetics, and diverse industrial sectors. They have long been regarded as highly effective broad‐spectrum antimicrobial agents with a relatively low risk of resistance development [73, 74]. However, in practical applications, formulations containing QACs frequently leave residues on surfaces without subsequent rinsing or neutralization. This results in prolonged exposure of microbial communities to subinhibitory concentrations [75]. Such conditions favor the survival and enrichment of strains that are only inhibited at higher minimum inhibitory concentrations (MICs), thereby promoting the selection and spread of resistant microbial populations [76, 77].

An increasing number of microbiological and epidemiological studies confirm reduced susceptibility of clinical and environmental isolates to quaternary ammonium compounds (QACs) [74, 78]. Research has demonstrated elevated minimum inhibitory concentrations (MICs) for QACs *in Staphylococcus aureus* [79], *Enterococcus species* [80], and various Gram‐negative pathogens compared with standard laboratory strains, particularly in settings involving surface disinfection or frequent disinfectant use [80, 81, 82]. Similar phenomena have been observed in bacteria isolated from food processing plants, livestock facilities, and wastewater treatment plants, indicating that adaptation to QACs is not confined to healthcare settings [83, 84]. In many such isolates, reduced QAC susceptibility co‐occurred with multidrug resistance to conventional antibiotics and the presence of mobile genetic elements carrying qacA/B, smr, and other resistance determinant clusters [84, 85], suggesting QAC exposure as a contributing factor to multidrug resistance development.

**FIGURE 4:**
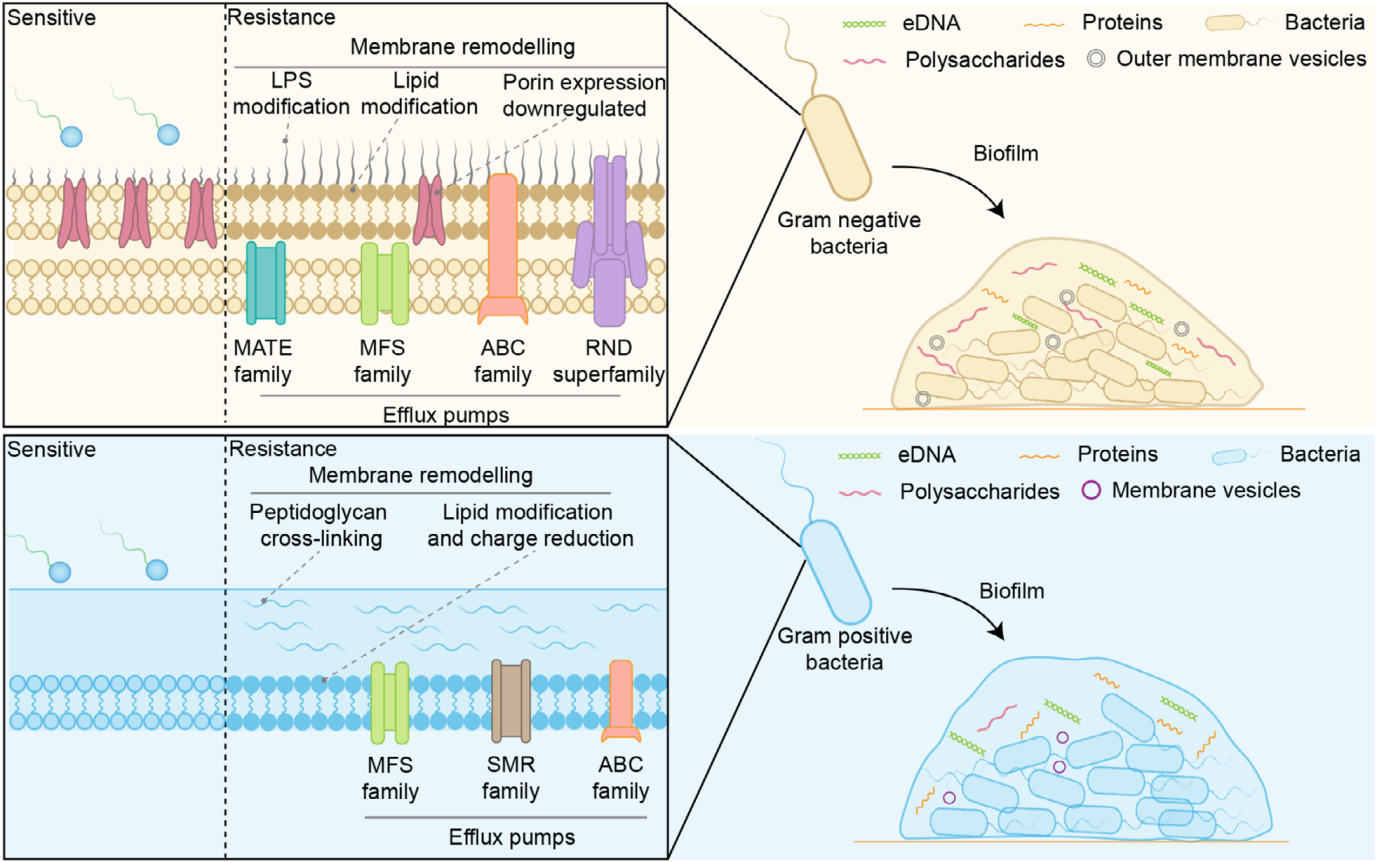
Bacterial Resistance to QACs comparison between Gram‐positive and Gram‐negative bacteria.

These observations collectively raise a critical question: How do bacteria adapt to quaternary ammonium compounds? Several distinct yet often overlapping mechanisms have been described, including active efflux of cationic biocides [86], remodeling of the cell envelope [87], and collective resistance within biofilms [88]. We will now summarize current knowledge regarding these resistance and adaptation mechanisms and explore how they contribute to the persistence of QAC‐resistant and multidrug‐resistant microbial communities across diverse environments (Figure 4).

### 5.1 Efflux Pumps

The extrusion pump systems encoded by genetic elements are key determinants for bacterial expulsion of QACs and antibiotics [89, 90]. These primarily include the major facilitator superfamily (MFS) [91, 92], ATP‐binding cassette (ABC) family [93, 94], small multidrug resistance (SMR) family [95, 96], resistance‐nodulation‐division (RND) superfamily [97, 98], multidrug and toxic compound extrusion (MATE) family [99], and proteobacterial antimicrobial compound efflux (PACE) family [100]. Efflux pumps are widely present in both Gram‐negative and Gram‐positive bacteria; however, due to differences in cell envelope structure, the primary efflux pump families and their contribution to quaternary ammonium compound (QAC) resistance vary significantly between these two bacterial groups.

In Gram‐negative bacteria, the presence of a double‐layered membrane structure has driven the evolution of powerful ternary RND systems that span both inner and outer membranes [97, 101]. For example, the AcrAB–TolC complex in *Escherichia coli* or the MexAB–OprM complex in *Pseudomonas aeruginosa* can capture quaternary ammonium compounds (QACs) and other toxic substances from the cytoplasmic membrane or periplasm and directly expel them into the external environment, effectively by passing the periplasmic space [102, 103]. These RND pumps, supported by other transporters from the MATE [99], MFS [104], and PACE families [105], typically result in reduced broad‐spectrum resistance and, when over‐expressed, lead to high‐level multidrug resistance. In clinical isolates, such efflux systems are often linked to integron or plasmid‐borne qacEΔ1 and antibiotic resistance genes [106, 107].

In Gram‐positive bacteria, the efflux of quaternary ammonium compounds (QACs) is primarily mediated by uniporters belonging to the MFS, SMR, and ABC families [108]. For example, *Staphylococcus* contains plasmid‐encoded MFS (QacA and QacB) as well as SMR (QacH, and QacJ) [109]. These transporters reduce bacterial susceptibility to various quaternary ammonium disinfectants but typically cause only a slight increase in minimum inhibitory concentration (MIC) [110]. However, under practical application conditions, even such minor MIC shifts can compromise disinfection efficacy if quaternary ammonium compounds persist on surfaces. Thus, although efflux‐mediated QAC resistance in Gram‐positive bacteria typically manifests as reduced susceptibility rather than high resistance [111], it plays a crucial role in the persistence of these microorganisms under biocide pressure.

### 5.2 Cell Membrane Remodeling

Cell membrane remodeling is a key adaptive mechanism for bacterial survival under QAC stress. In Gram‐negative bacteria, including *Escherichia coli* and *Pseudomonas aeruginosa*, prolonged exposure to QACs induces alterations in lipopolysaccharide structure, downregulation of porins, and increased membrane rigidity. These changes are primarily regulated through the mar‐sox‐rob and RpoE pathways [112, 113, 114]. In Gram‐positive bacteria, such as *Staphylococcus aureus* and *Enterococcus faecalis*, exposure to sublethal concentrations of QACs induces alterations in membrane lipid composition, surface charge, and peptidoglycan cross‐linking [115, 116, 117]. Collectively, these changes reduce electrostatic attraction and permeability to cationic disinfectants. These responses are typically mediated by envelope stress regulators, resulting in a reduced susceptibility rather than a highly resistant phenotype. Such structural modifications reduce QACs accumulation and enhance efflux‐mediated defense mechanisms, thereby promoting bacterial survival under bactericidal stress. In many studies, membrane adaptation to quaternary ammonium exposure has been inferred primarily through transcriptomic changes or indirect biophysical measurements, rather than through comprehensive lipidomics or high‐resolution structural analysis. Therefore, the causal relationship between specific remodeling events and specific levels of quaternary ammonium tolerance remains incompletely understood.

### 5.3 Collective Resistance Within Biofilms

Biofilms provide a protective layer at the population level, significantly reducing the effectiveness of QACs. Bacteria embedded in extracellular polymeric substances (EPS) matrices are generally much less sensitive to QACs than planktonic bacteria, often requiring several times higher QAC concentrations or longer contact times to achieve a similar logarithmic reduction in viable counts. Negatively charged and chemically heterogeneous EPS can chelate cationic QAC molecules, locally neutralizing their bactericidal activity before they reach deeper biofilm layers. Simultaneously, nutrient and oxygen restriction within the biofilm promotes the growth of slow‐growing or dormant persistent cells, which are less affected by membrane‐active substances. In addition, biofilms could also promote or upregulate efflux pumps or other genetic mutations in bacteria resulting in physiological changes related to bactericidal sensitivity.

Gram‐negative and Gram‐positive bacteria also exhibit differences in biofilm‐mediated tolerance to QACs. In Gram‐negative pathogens such as *Pseudomonas aeruginosa* and *Acinetobacter baumannii*, the double‐layered cell envelope combines with a hydrated biofilm rich in anionic extracellular polysaccharides and extracellular DNA [118, 119]. This structure forms a multilayer barrier where EPS slows QAC penetration and partially neutralizes cationic molecules, while RND efflux pumps expel residual QACs [120, 121, 122]. Consequently, biofilm‐forming Gram‐negative bacteria can exhibit significant broad‐spectrum reduction in drug susceptibility.

Gram‐positive bacteria, such as Staphylococcus and Listeria monocytogenes, lack an outer membrane but possess a thick peptidoglycan layer coated with teichoic acids on its surface [123]. Their biofilm EPS, rich in teichoic acids, proteins, and extracellular DNA, forms a negatively charged barrier capable of binding and sequestering QACs [124, 125]. Therefore, biofilm formation and surface charge shielding are primary factors in QAC tolerance, while efflux typically results only in minor increases in MIC [125, 126, 127]. Thus, despite differing underlying structures, biofilms of both Gram‐negative and Gram‐positive bacteria create unique physicochemical and genetic environments that enhance resistance to quaternary ammonium compounds compared with planktonic cultures. However, most existing data comes from static in vitro biofilm models, while real‐world application environments involve concentration fluctuations, complex organic contamination, and diverse microbial communities. Therefore, further clinical studies are needed to quantitatively predict the performance and failure rates of QAC‐based disinfection protocols in practical applications.

Overall, existing evidence indicates that reduced bacterial susceptibility to quaternary ammonium compounds (QACs) rarely stems from a single dominant mechanism. Instead, efflux‐mediated MIC changes, cell envelope remodeling, and biofilm‐associated tolerance often act synergistically, with their relative importance varying significantly across species, strains, and QAC structures [128, 129]. Another complication arises from the fact that many studies assess resistance based solely on MIC values for planktonic states, which fail to reflect the spatiotemporal gradients and population effects present in real‐world disinfection scenarios. Addressing these conceptual and methodological shortcomings is crucial for rationally designing next‐generation QACs and their application strategies that minimize the risks of resistance development and co‐selection of multidrug‐resistant organisms.

## 6 Outlook

As described above, traditional monomeric quaternary ammonium compound (QAC) disinfectants exhibit numerous limitations, including reduced sensitivity mediated by efflux pumps, membrane remodeling, and biofilm‐associated tolerance, as well as environmental persistence and potential co‐selection of multidrug‐resistant strains. To face these challenges, recent research has shifted toward more complex QAC‐based structures. Overall, four design directions can be distinguished: QAC‐based surface materials providing persistent antimicrobial activity [130, 131]; QAC‐derived systems with enhanced biocompatibility for targeted drug delivery [132, 133, 134]; biobased degradable QACs designed to reduce environmental persistence and selection pressure [135, 136, 137]; and multifunctional systems combining QAC moieties with other antimicrobial approaches, including metal nanoparticles [138, 139, 140], antimicrobial peptides [141, 142, 143], and photodynamic therapy [144, 145, 146].

### 6.1 QAC‐Based Surface Materials

QACs solutions are easily diluted and prone to deactivation by organic contamination, leading to repeated microbial exposure at subinhibitory concentrations. This favors the selection of bacterial populations insensitive to QACs. In contrast, QAC‐based surface materials maintain high densities of cationic groups at the solid–liquid interface, enabling effective contact‐mediated killing of adherent bacteria while reducing the need for repeated recoating. Such coatings have been studied for diverse applications including medical devices, wound dressings, textiles, food contact materials, and hightouch hospital infrastructure, where sustained inhibition of microbial colonization is particularly critical.

Quaternary ammonium compound‐based surface materials can be categorized in two primary design principles. The first principle involves covalently grafting of quaternary ammonium groups onto polymer backbones or inorganic surfaces to form low‐leakage coatings that exert their antimicrobial activity upon contact [130, 130]. Examples include acrylic and polyurethane coatings with quaternary ammonium side chains, quaternary ammonium‐grafted silica or glass, and polymer brushes featuring high‐density cationic chains extending into the aqueous phase [147, 148, 149, 150]. These surfaces typically disrupt bacterial cell membranes upon contact, inhibiting initial adhesion and subsequent biofilm formation [151]. The second approach involves embedding or incorporating quaternary ammonium salts into ionic liquids or crosslinked polymer networks [152, 153, 154]. These materials combine contact‐mediated bactericidal activity with sustained, controlled release of low concentrations of quaternary ammonium salts. By adjusting parameters such as alkyl chain length, counterions, crosslinking density, and hydrophilicity, quaternary ammonium salt‐based ionic liquids and polymer matrices can be engineered to balance antimicrobial efficacy, mechanical stability, and fouling resistance [155, 156].

Despite the promising prospects of QAC‐based surface materials, several issues remain unresolved. While high surface charge density promotes rapid bactericidal activity, it may also enhance cytotoxicity, hemolysis, and pro‐inflammatory responses [157, 158, 159]. Surface aging, incomplete coverage, and the slow leakage of low‐molecular‐weight fragments may still result in QAC exposure concentrations below the inhibitory threshold under practical conditions, thereby allowing resistance to develop [151, 160]. Furthermore, the long‐term performance of QAC surfaces under mechanical wear, repeated cleaning, UV exposure, and complex organic fouling remains inadequately characterized [161, 162, 163]. Most testing protocols also overlook the presence of mixed‐species biofilms. Therefore, more systematic, standardized, and field‐based evaluations are urgently needed to determine whether QAC surface materials genuinely overcome the limitations of traditional disinfectants.

### 6.2 QAC‐Derived Systems with Enhanced Biocompatibility

QAC‐derived cationic surfactants have been designed as components of drug delivery systems. Their permanent positive charge enables them to efficiently complex with anionic carriers such as nucleic acids and promotes interactions with negatively charged cell membranes [164, 165]. To mitigate the nonselective membrane disruption and hemolysis associated with conventional QAC disinfectants, recent research has focused on developing more biocompatible structures, including QAC‐based cationic lipids and amphiphilic surfactants that can be assembled into liposomes or micelles, QAC functionalized polymers (e.g., quaternized chitosan or polymethacrylates), and amphiphilic block copolymers or prodrug conjugates with QAC groups [166, 167, 168, 169]. These materials are often used in conjunction with targeting ligands or stimulus‐responsive linkers to improve tissue selectivity. However, in many formulations, the therapeutic window between effective delivery and unacceptable cytotoxicity remains narrow, necessitating more systematic in vivo and long‐term safety studies to determine the biocompatibility of QAC‐derived carriers.

### 6.3 Biobased QACs

Biobased degradable quaternary ammonium compounds (QACs) are considered a complementary strategy that holds promise for addressing the persistent presence of traditional QACs in the environment and their co‐selectivity for antibiotic resistance. The cationic head group and/or hydrophobic framework are derived from renewable structural units, such as amino acids, sugars, choline derivatives, or other biomass feedstocks, and easily cleavable linking groups (e.g., esters, carbonates, or amides) are introduced into the hydrophobic tail or polymer backbone to promote abiotic and enzymatic degradation [34, 170, 171, 172, 173]. This design aims to maintain antimicrobial activity during use but degrade more rapidly and reduces accumulation once released into wastewater treatment systems or the natural environment. However, balancing antimicrobial efficacy and degradability is extremely challenging. Increasing hydrophobicity and charge density generally improves bactericidal activity but often slows biodegradation and increases toxicity to nontarget organisms [174, 175], while highly unstable or strongly hydrophilic designs may not achieve sufficient bactericidal effects in practical applications [176, 177]. Therefore, current research provides some promising proof‐of‐concept examples, but a systematic study of the relationship between structure, antimicrobial activity, and degradability is still needed.

### 6.4 Multifunctional QAC Systems

To overcome the limitations of single membrane disruption mechanisms, QACs are being integrated into multifunctional hybrid systems in combination with other antibacterial mechanisms. One research direction involves coupling QAC groups with metal nanostructures such as silver and copper [138, 178, 179]. The membrane‐targeting cationic groups enhance bacterial binding and biofilm penetration, while metal ion release or redox activity provides additional bactericidal mechanisms. Another category is photodynamic QAC constructs, where photosensitizers are modified with quaternary ammonium salt groups or loaded onto quaternary ammonium salt‐based carriers [180, 181, 182, 183]. This leads to the generation of photo triggered reactive oxygen species near the bacterial membrane, thereby enhancing the killing effect against drug‐resistant and multidrug‐resistant strains. While these composite systems utilize complementary mechanisms and may reduce the likelihood of developing resistance to any single component, they also increase structural and formulation complexity, raising concerns about cumulative toxicity, such as metal ion exposure, immunogenicity, or photodamage [141, 183, 184].

## 7 Summary

The use of quaternary ammonium surfactants as general and medical disinfectants emerged in the beginning of the 20th century. Their general structure is described as NR_4_^+^ in which R is modulated in several ways to optimize and fine tune their biological potential. The mechanism of action can be briefly divided into two steps: (i) the adhesion to the cellular membrane and (ii) the insertion into the cellular membrane. Thereby, they induce a complete solubilization of the bacteria's outer cell surface. The biological activity is mainly governed by the chain length of the hydrophobic tail, with a cut‐off edge between 12 and 18 carbon atoms and a proper balance between the hydrophobic and hydrophilic units. Interestingly, evidence has been found that bacterial growth inhibition must not necessarily result from a complete membrane disruption but can also arise from interaction with intracellular structure and charge neutralization. Supramolecular aggregates show a promising approach to induce biological selectivity for bacterial cells over mammalian cells. Unfortunately, reduced QAC susceptibility has been found in clinical and environmental bacterial isolates. Resistance can occur through genetic modifications encoding for (i) efflux pumps that expel toxic compounds from the cytoplasm into the external environment and (ii) changes in the cell membrane including alterations in the LPS structure, downregulation of porins and an increased membrane integrity to reduce electrostatic attraction and permeability. Intrinsic bacterial resistance especially associated to biofilm formation. The biofilms extracellular polymeric substance forms a protection barrier that quenches the biological activity of QACs. To overcome these limitations, new trends in the design of quaternary ammonium based cationic materials have emerged including: (i) QACs surface coating to avoid the overuse of QAC‐based disinfectants, (ii) cationic lipids, cationic polymers, and (iii) biobased materials (sugars, amino acids) as cationic head groups to improve selectivity of bacteria cells over mammalian cells, followed by (iv) multifunctional QACs that combine QACs biological activity with for example antimicrobial activity of redox active metal nanostructures to improve the long‐term antibacterial activity.

## Funding

This study was supported by the Deutsche Forschungsgemeinschaft (431232613 – SFB 1449 and IRTG 2662 (Project ID: 434130070)), Werner Siemens‐Stiftung, and Joachim Herz Stiftung.

## Conflicts of Interest

The authors declare no conflicts of interest.
